# Dataset knowledge, attitude, and trust of Indonesian selected public group toward agribiotechnology application

**DOI:** 10.1016/j.dib.2020.106496

**Published:** 2020-11-04

**Authors:** Rachmat Hidayat, Tree Setiawan Pamungkas, Lukman Wijaya Baratha, Ahmad Munif Mubarok

**Affiliations:** Faculty of Social and Political Science, University of Jember, East Java, Indonesia

**Keywords:** Agribiotechnology, Knowledge, Attitude, Trust

## Abstract

The focus of this research is to present an analysis of the knowledge, attitude, and trust of selected Indonesian public groups regarding the application of Agribiotechnology. This study employs a descriptive research design. The data consists of 266 respondents in two different localities in east Java, Indonesia: Jember and Bondowoso. Eight different categories of respondents were defined: students, scientists, non-government organizations, media, policymakers, consumers, producers and religious scholars.  Participants responded to items assessing their knowledge, attitudes and trust toward the use of agribiotechnology, specifically in food production and how their cultural ethics, norms, or religious beliefs influence their engagement with the technology. The findings highlighted varying perspectives on the knowledge, attitude, and trust among the eight groups towards agribiotechnology application concerning responses that emphasized several content areas such as the genetic modification of crops and plants and implications of the technology on the daily lives of the Indonesian.

## Specifications Table

**Table:** 

Subject Area	Social Science
More Specific Subject Area	Public Policy, Sociology, Biotechnology, Agribiotechnology
Type of Data	Table, Figure
How Data Was Acquired	Questionnaire
Data Format	Raw, analyzed
Experimental Factors	The sample was divided into eight different selected public groups: students, scientists, non-government organizations, media, policymakers, consumers, producers, and religious scholars. These selected groups were purposely chosen based on their representation as a public group.
Experimental Feature	Knowledge, attitude, and trust are critical to the success for the adoption of agribiotechnology product (genetic modification of crops and plants) in the daily lives of an individual or communities in any given country.
Data Source Location	The data was obtained from two regencies in East Java: Jember and Bondowoso, Indonesia.
Data Accessibility	Data is included in this article.

## Value of the Data

• The dataset provides insight into the elected public group in Indonesia on their perception of knowledge, attitude, and trust toward the application and the adoption of agribiotechnology products in their daily lives.
• The dataset partial analysis could explain the relationship between knowledge, attitude, and public trust toward agribiotechnology application and adoption.
• The dataset on this paper could be used by the greater community of scientists and academics in the field of Agribiotechnology or any other related major with an emphasis on the advancement of public trust for agribiotechnology products.
• The dataset will help stakeholders of agribiotechnology development such as companies, policymakers, scientists, farmers, and communities to develop a useful model for mainstreaming the adoption of agribiotechnology products in the context of developing countries.

## 1 Data Description

The dataset mainly consists of the perceptions of the selected public group in two regencies of Jember and Bondowoso, within the localities of Horseshoe area (*Tapal Kuda*) in East Java, Indonesia, on (1) Knowledge (2) Attitude and (3) Trust on agribiotechnology application and products. Table 1 provides respondent demographic distribution. The distributions of 266 respondents categorized by Fifteen percent of participants were college students, 10.2% worked at non-government organizations, 15% were scientists, 13.2% were producers, 9.8% worked for the media, 15% were consumers, 10.2% were policymakers, and the last 11.7% were religious scholars (Table 1). The distribution of respondents by gender; 163 (61,28%) were male, while 103 (38,72%) were female.

**Table 1:** Demographics table.

*N* = 266		Frequency	Percent
Gender	Male	163	61.28%
	Female	103	38.72%
Location	Jember	144	54.14%
	Bondowoso	122	45.86%
Age	17–26	80	30.08%
	27–36	56	21.05%
	37–46	57	21.43%
	47–56	42	15.79%
	57–66	28	10.53%
	67–76	3	1,13%
Category	College Students	40	15.04%
	NGO	27	10.15%
	Scientist	40	15.04%
	Producer	35	13.16%
	Media	26	9.77%
	Consumer	40	15.04%
	Policy Maker	27	10.15%
	Religious Scholar	31	11.65%

Note: the four (4) of the demographic variable coded in data as Gender (1-Male, 2-Female), Location(1-Jember, 2-Bondowoso), Age (1 For 17–26, 2 for 27–36, 3 for 37–46, 4 for 47–56, 5 for 57–66) Category (1-Student College, 2-NGO, 3 Scientist, 4-Producer, 5-Media, 6-Consumer, 7-Policy Maker, 8-Religious Scholar).

The distribution of gender shows that a larger percentage of the men were actively engaging in the agribiotechnology activities at local level. The distribution of respondents by location; 144 (54,14%) from Jember; and 122 (45,86%) form Situbondo. The respondents distribution by ages category; Age 17–26 (30,08%), Age 27 – 36 (21,05%), Age 37–46 (21,43%), Age 47–56 (10,53%) and Age 57–66 (1,13%).

Table 2 provides information on the validity of the variable and factor loadings. The knowledge variable includes nine factors, the attitude variable includes nine factors, and the trust variables include seven factors. Each item loads on the knowledge variable between 0,580 and 0,891. Each item on the attitude variable has a loading between 0,421 and 0,697. Each item on trust variable loading between 0,384 and 0,625. Overall, KMO and Bartlett's Test Value also suggest the suitability of structure detection.

**Table 2:** Factor loading and validity.

Variables	Code	Factor Loading		
Knowledge	Kn1	0.697		
	Kn2	0.715		
	Kn3	0.656		
	Kn4	0.794		
	Kn5	0.766		
	Kn6	0.699		
	Kn7	0.891		
	Kn8	0.621		
	Kn9	0.580		
Attitude	Att1		0.443	
	Att2		0.421	
	Att3		0.582	
	Att4		0.430	
	Att5		0.571	
	Att6		0.660	
	Att7		0.697	
	Att8		0.469	
	Att9		0.660	
Trust	Tru1			0.593
	Tru2			0.428
	Tru3			0.441
	Tru4			0.625
	Tru5			0.628
	Tru6			0.384
	Tru7			0.542
		Kaiser-Meyer-Olkin Measure of Sampling Adequacy.	0.866	
		Bartlett's Test of Sphericity	Approx. Chi-Square	2241.991
			df	300
			Sig.	0.000

Note: Kn (Knowledge), Att (Attitude), Tru (Trust).

**Table 3:** The one-way Anova test.

	Anova	
	Sig	Mean
Kn1	0,130	2,62
Kn2	0,028	2,38
Kn3	0,001	2,70
Kn4	0,505	1,64
Kn5	0,040	3,05
Kn6	0,103	2,97
Kn7	0,124	3,44
Kn8	0,000	2,62
Kn9	0,030	2,39
Att1	0,001	1,92
Att2	0,000	2,16
Att3	0,008	2,26
Att4	0,000	2,53
Att5	0,000	2,39
Att6	0,001	3,02
Att7	0,095	1,35
Att8	0,004	2,37
Att9	0,098	2,73
Tru1	0,000	2,17
Tru2	0,001	1,40
Tru3	0,000	1,31
Tru4	0,005	1,61
Tru5	0,193	1,45
Tru6	0,004	1,40
Tru7	0,266	1,62

## 2 Experimental Design, Materials and Methods

The dataset mainly consists of three parts: (1) Knowledge (2) Attitude and (3) Trust. These three components are critical to the success and acceptance of agribiotechnology, particularly regarding the commercial development of genetically modified crop plants and food production [3]. In line with global efforts to eliminate starvation and to improve food security, agribiotechnology is curiously considered unfavorable in most of the developing countries in African regions [2]. Providing a good recommendation for fostering, mainstreaming, and creating a public trust for the stakeholder of agribiotechnology development is crucial to the successful adoption of Biotechnology domestically and internationally among all groups in the community [4,5].

The data was collected by in-person surveys of adults in the selected public groups in the East Java Province, Indonesia. Since membership in the selected public groups was not publicly available, participants were recruited using a stratified purposive sampling technique. Although the sample chosen using this technique may not entirely reflect Indonesia's actual population, this technique allows respondents from different public groups that are traditionally underrepresented in random sampling techniques.

Moreover, consent was obtained from each participant for their participation. Questionnaires were constructed from references [1,^2^,^4^,5] and available on the supplementary material section. Research participants register their responses to items on a Likert scale, ranging from 1 (very weak) to 5 (very strong). Composite variables for knowledge, attitudes, and trust were created by averaging the items of those scales.  Higher items scores reflected more knowledge, more positive attitudes, and greater trust in agribiotechnology. Data analysis achieved by SPSS (v25) by performing KMO and Bartlett's Test for factor loading and sampling adequacy value and dan One-Way ANOVA test for correlation value.

The One-way ANOVA test on the Knowledge group variable consists of 9 factors (Kn1-Kn9) (Table 3) result in a range of mean scores from 1,64 to3,44, with a significant score range between 0,00 to 0,505, which means that not every factor of knowledge on the application of agribiotechnology Indonesia correlates to the Indonesian selected public groups' perspectives on agribiotechnology.

Further, Table 3 illustrates a significant correlation between Indonesian selected public groups' attitudes towards agribiotechnology application and adoption. This means each group posses a variably determining factor on the adoption and application of agribiotechnology products in their daily lives. This correlation appears on the mean score, ranging from 1, 92 to 3,02, with a significant score ranging from 0,000 to 0,098.

Further, the trust variable achieved a range of mean scores between 1,31 to 2,17, with a significant score ranging from 0,000 to 0,098. This implies that the group variable significantly influences the Indonesian selected public groups' trust toward the adoption and the application of agribiotechnology. This dataset implies further analysis of whether knowledge, attitude, and trust are a determinant factor for the people's adoption and application of Agribiotechnology in a broader community or state context.

## Ethics Statement

A plain language statement is available during the research and containing information on the research topics on knowledge, attitude, and trust of Indonesian public groups regarding the application of Agribiotechnology. Potential research participants were asked to read the plain language statements and complete a research consent form if they are willing to participate in this project voluntarily. For publication, the researcher uses direct quotes and provides a pseudonym to protect participants' anonymity when giving any information that would refer directly to the participants /identify particular participants.

## Declaration of Competing Interest

The researcher does not have a conflict of interest with various parties in presenting the research data, and no party intervenes in the research results.

## Appendix Supplementary Materials

Image, application 1

Image, application 2

Image, application 3

Image, application 4
