# Supplementary material for: Dataset knowledge, attitude, and trust of Indonesian selected public group toward agribiotechnology application
Source: Data Brief. 2020 Nov 4;33:106496. doi: 10.1016/j.dib.2020.106496 (PMC7666304; doi:10.1016/j.dib.2020.106496)

**Agrobiotechnology Products Aplication and Adoption for Daily Lives Survey**

Thank you for agreing to take part in this important survey measuring the Knowledge, Attitude, and Trust of Indonesian Selected Public Group Toward Agribiotechnology Application. This survey only take 5-10 minutes to complete and please be assured to fill your identitiy and provide the question with an answer. All of your answer will be treated with strictest confidentiality

1. **Credentials of the respondents**

**(Please list your name, age, job, adress, phone here)**

**Instruction:**

please pick a number to represent your agreement or your disagreement to the following statement bellow


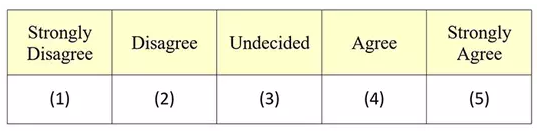


**Question**

1. **Knowledge on Agrobiotechnology Application and Adoption**
2. DNA or genus of organisms such as micro organisms, plants and animals, including human genes have been mapped


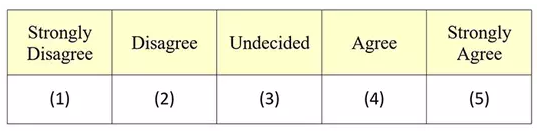


1. The food you consume contains DNA or genes from plants or animals


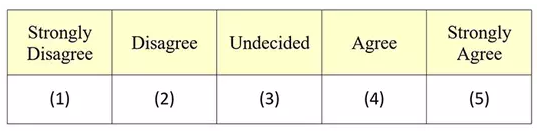


1. There is presences in the market of medication that made by agricultural biotechnology products.


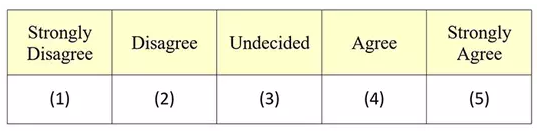


1. Every product that is circulating in the community and based on biotechnology products needs to be assessed based on the religious aspects of the community


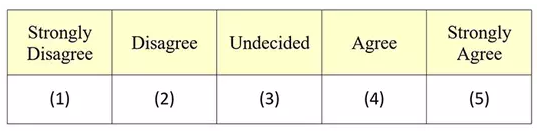


1. Genetic engineering have been used in almost all aspects of agriculture practice in the region of tapal kuda (Horsehoe region) of east java area?


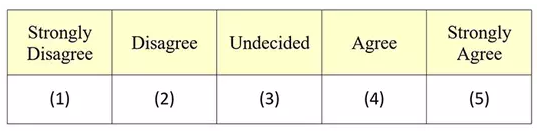


1. Foods that you consume every day containing a products from genetic engineering


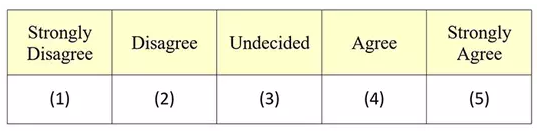


1. There are no government regulation that regulates genetic engineering and the circulation of agricultural biotechnology products


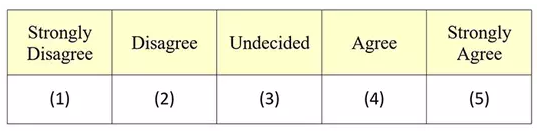


1. The Indonesian government in recent decades has encouraged and expanded the use of genetic engineering in the agricultural sector?


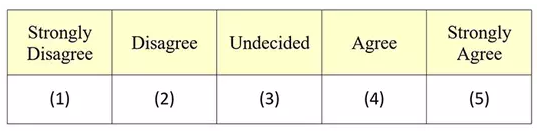


1. For the longer term and broader aspects, genetic engineering will improve the standard of living of your community


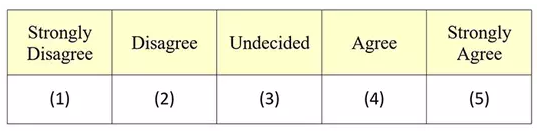


1. **Attitude on Agrobiotechnology Application and Adoption**
2. The development of science in the field of agricultural biotechnology on tapal kuda (Horsehoe region) of east java will drive progress for the community


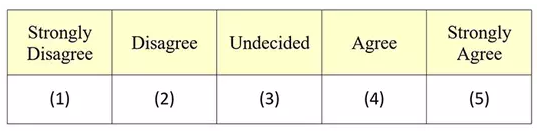


1. Genetic engineering on agricultural biotechnology will encourage growth and attempt for strengthening food security in the tapal kuda (Horsehoe region) of east java


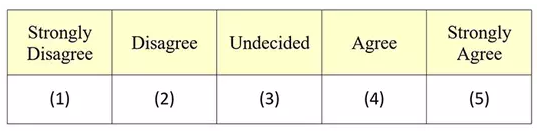


1. Agricultural bioctechnology will reduce the impact of pesticide use and environmental damage


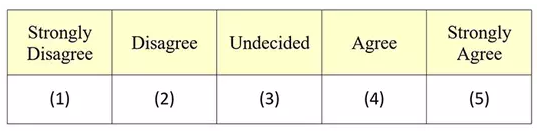


1. Agricultural bioctechnology will improve healthy and safe food products


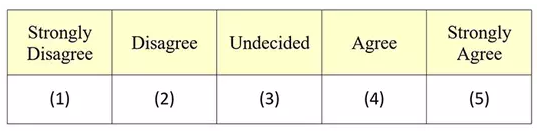


1. Genetic engineering will increase the variety of seeds and agricultural products that are compatible with the local ecosystem


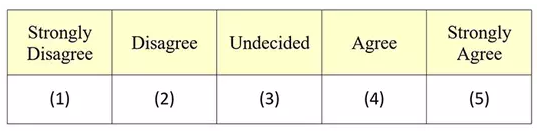


1. Agricultural biotechnology-based food and beverage products possesed DNA map information that can be known and open for the public


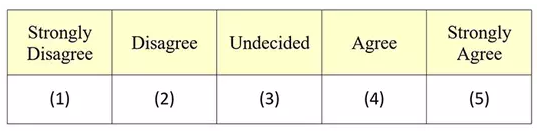


1. Agricultural biotechnology must be not contrary to the values of religions and community


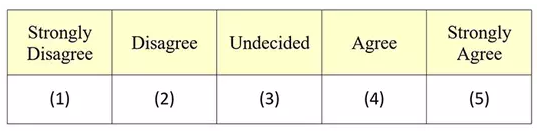


1. Agricultural biotechnology will increase the nutritional value of the food


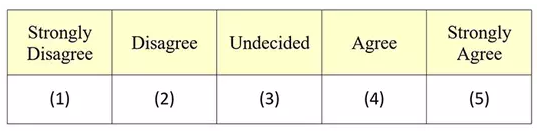


1. Agricultural biotechnology will reduce the cost of food production


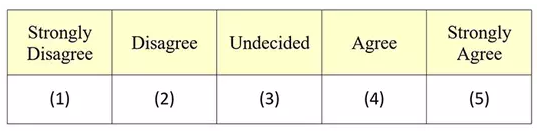


1. **Trust on Agrobiotechnology Application and Adoption**
2. Agricultural biotechnology products and application is unavoidable, most people use it


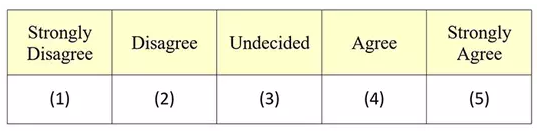


1. Genetic engineering in the domain of agricultural sector requires open socialization from the government?


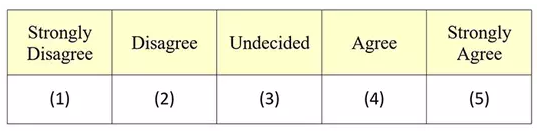


1. Agricultural biotechnology companies have responsibility for the products they produce to society at large


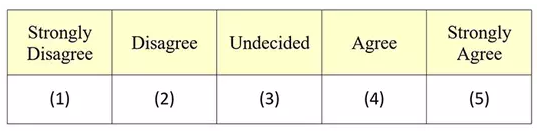


1. Genetic engineering in the agricultural sector requires open support from the community


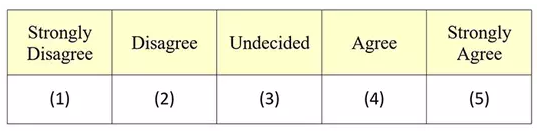


1. Agricultural biotechnology must reduce adverse effects on the environment and public health


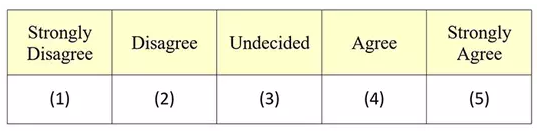


1. Cooperation between sectors in the community and the government is needed in managing and overseeing the development of agricultural biotechnology


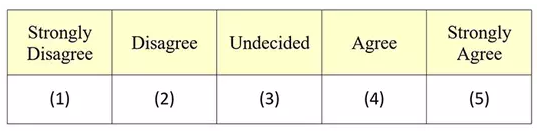


1. Society is the first sector to impact the positive and negative outcome of agricultural biotechnology. So that their role and articulation must be facilitated through the presence of other community or social institutions


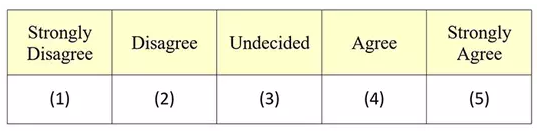

Supplement: Supplementary file 3 [file mmc3.docx]
